# An Intelligent Micromachine Perception System for Elevator Fault Diagnosis

**DOI:** 10.3390/mi17040401

**Published:** 2026-03-25

**Authors:** Li Lai, Shixuan Ding, Zewen Li, Zimin Luo, Hao Wang

**Affiliations:** 1 Guangdong Institute of Special Equipment Inspection and Research Huizhou Branch, Huizhou 516001, China; dingshixuan@gdsei.org.cn (S.D.); lizewen@gdsei.org.cn (Z.L.); luozimin@gdsei.org.cn (Z.L.); 2 School of Cyber Engineering, Xidian University, Xi’an 710126, China; wanghao@xidian.edu.cn

**Keywords:** MEMS, efficient AI, anomaly detection, edge computing, agent

## Abstract

Elevator fault diagnosis heavily relies on high-precision sensing of microscopic physical states. Although Micro-Electro-Mechanical System (MEMS) sensors can capture such subtle features, they are constrained by high-frequency data streams, environmental noise, and the semantic gap between raw sensor data and actionable maintenance decisions. This study proposes a collaborative edge–cloud intelligent diagnosis framework specifically designed for elevator systems. On the edge side, a lightweight temporal Transformer model, ELiTe-Transformer, was designed and deployed on the Jetson platform. This model enhances sensitivity to event-driven MEMS signals through an industrial positional encoding mechanism and by integrating linear attention and INT8 quantization techniques, achieving a real-time inference latency of 21.4 ms. On the cloud side, retrieval-augmented generation (RAG) technology was adopted to integrate physical features extracted at the edge with domain knowledge, generating interpretable diagnostic reports. The experimental results show that the overall accuracy of the system reaches 96.0%. The edge–cloud collaborative framework improves the accuracy of complex fault diagnosis to 92.5%, and the adoption of RAG reduces the report hallucination rate by 71.4%. This work effectively addresses the bottlenecks of MEMS perception in elevator fault diagnosis, forming a closed loop from micro-signal acquisition to high-level decision support.

## 1. Introduction

With the increasing integration of the Industrial Internet of Things (IIoT) and intelligent maintenance technologies, the fusion of high-precision perception and real-time decision-making has become crucial for ensuring the safe operation of complex electromechanical systems [1,2,3]. Elevators, as indispensable vertical transportation systems in high-rise buildings, directly impact public safety with their operational reliability [4]. However, traditional manual inspections and periodic maintenance methods are inefficient, struggle to capture subtle hardware degradation trends during operation, and are hampered by slow response times when dealing with sudden failures. With the advancement of sensor technology, data-driven intelligent fault diagnosis has become a research hotspot [5]. However, establishing a complete closed loop from physical signals to maintenance decisions remains a significant challenge.

Micro-Electro-Mechanical System (MEMS) sensing technology integrates mechanical elements, sensors, and electronic circuits onto a single chip through micron-scale fabrication processes, offering advantages such as small size, low power consumption, fast response, and ease of mass production [6]. It has become a core sensing means in the field of industrial monitoring [7,8,9]. In elevator fault monitoring, MEMS-based sensors like accelerometers and microphones can capture microscopic physical changes difficult for traditional macro-sensors to detect, such as specific frequency vibrations from early bearing wear or subtle jitter caused by micron-level guide rail irregularities [10,11]. Common elevator fault types can be categorized into mechanical component wear (such as bearing wear and guide shoe wear), drive system anomalies (e.g., traction machine faults and motor current imbalance), guide rail degradation (e.g., uneven rail joints and deformation), door system sticking, and faults induced by environmental factors (e.g., pit water accumulation and temperature and humidity exceeding limits). In MEMS sensor monitoring, these faults manifest as specific physical indicators: for example, early-stage bearing wear can be identified by a vibration acceleration envelope peak >0.12 gE; traction machine misalignment or rotor imbalance can be diagnosed by a vibration velocity RMS > 3.5 mm/s; uneven guide rails can cause the vertical vibration peak inside the car to exceed 0.6 m/s^2^; guide shoe wear is reflected by a horizontal lateral vibration A95 value exceeding 0.25 m/s^2^; door system sticking is often accompanied by an abnormal sound pressure level increase exceeding 15 dB; electrical faults, such as motor inter-turn short circuits, manifest as a three-phase current unbalance exceeding 10%; inverter faults can be detected by a total voltage harmonic distortion rate exceeding 5%; the risk of pit water accumulation is monitored by a water level sensor with a threshold set at 50 mm; and environmental factors like pit humidity exceeding 85% trigger an environmental alert.

However, MEMS perception systems face a series of inherent limitations in practical industrial deployment, hindering their transition from laboratory accuracy to field usability. Through in-depth analysis of the elevator monitoring scenario, we identify three core limitations of MEMS perception systems in industrial applications. (1) The conflict between data throughput and computational power: The high sampling frequency of MEMS sensors generates massive data streams, imposing stringent requirements on the real-time processing capabilities of edge devices. Traditional cloud-based solutions fail to meet real-time needs due to transmission latency [12]. (2) Signal non-uniformity and noise interference: In industrial environments, robustness to noise, sensor drift, and varying operating conditions is essential for the practical deployment of fault diagnosis systems [13]. However, models trained under laboratory conditions often experience significant performance degradation when applied to real-world scenarios [14]. Environmental noise, electromagnetic interference in industrial settings, along with inherent bias drift and thermal noise of MEMS devices can easily submerge weak fault features [15]. Furthermore, critical fault signals are often non-uniformly distributed over time [16], contradicting the uniformity assumption of standard time-series models. (3) The semantic gap between numerical output and business decisions: MEMS sensors only provide physical quantity values such as voltage and frequency, lacking semantic explanations for root causes or the ability to generate specific repair suggestions. This makes it difficult for maintenance personnel to translate monitoring data into executable decisions.

Existing research methods have obvious shortcomings in addressing the above limitations. Traditional machine learning methods, such as Support Vector Machines (SVMs) [17] and multi-attribute decision making [18], struggle to handle the long-sequence characteristics and multi-modal correlations of MEMS data. Deep time-series models like LSTM [19] and the TCN [20] can capture certain temporal dependencies but suffer from gradient vanishing or limited receptive fields when processing ultra-long sequences as well as from insufficient robustness [21], and their computational efficiency often fails to meet edge deployment requirements. The standard Transformer architecture [22], while possessing powerful long-sequence modeling capabilities, has high computational complexity and a uniform positional encoding mechanism. This makes it unsuitable for resource-constrained edge environments and ineffective at capturing the event-driven nature of industrial data. Additionally, most existing diagnostic systems stop at fault classification, lacking the ability to combine physical evidence with domain knowledge for root cause analysis and repair suggestion generation. In industrial intelligent diagnosis, interpretability is critical to credible and applicable diagnostic conclusions. Zhao [23] argues that model parameters must carry clear physical or statistical significance to realize the mapping from data features to system states.

To systematically address these challenges, this study proposes an industrial diagnostic framework that deeply integrates edge computing with MEMS perception systems. At the edge side, the framework incorporates a lightweight time-series Transformer model named ELiTe-Transformer. It employs an industrial positional encoding mechanism to adapt to the event-driven characteristics of MEMS data and utilizes linear attention and INT8 quantization techniques for efficient inference, directly processing high-frequency data streams from multiple MEMS sensors. In the cloud, retrieval-augmented generation (RAG) technology is introduced to construct a professional knowledge base that integrates industry standards, technical manuals, and historical cases. It converts low-level MEMS sensor signals into physically grounded, traceable, and actionable maintenance decisions, thus bridging the semantic gap. Through edge–cloud collaborative framework, the framework ensures millisecond-level real-time response capability while achieving expert-level diagnostic depth, comprehensively addressing the various limitations of MEMS perception systems in industrial applications.

The main contributions of this paper are as follows:

This work identifies three critical gaps in industrial MEMS perception deployments: data throughput versus computing power, noise versus feature extraction, and value versus decision-making. Accordingly, an end-to-end edge–cloud collaborative framework diagnostic framework was constructed, rather than simply stacking algorithms.

We propose ELiTe-Transformer, a novel lightweight temporal model tailored for the event-driven nature of MEMS data. Diverging from the uniform position encoding of standard Transformers, we designed industrial positional encoding, which physically enhances the model’s sensitivity to weak fault signals. This enables millisecond-level real-time inference on the Jetson edge platform.

We innovatively applied RAG technology to bridge the data-to-decision gap. Extending beyond using LLMs solely for text-based Q&A, we constructed an elevator fault feature-augmented RAG agent. This agent can deeply integrate multi-dimensional MEMS feature chains extracted at the edge with structured domain knowledge to automatically generate reports containing root cause analysis and maintenance recommendations.

This work yielded a deployable intelligent maintenance agent. The research was not confined to simulation; it was validated on real-world elevator datasets and Jetson hardware, providing a complete intelligent operational maintenance solution for industrial MEMS perception.

The remainder of this paper is structured as follows: Section 2 reviews related work; Section 3 details the system design and methodology; Section 4 presents experimental results and discussion; Section 5 concludes the paper and outlines future research directions.

## 2. Related Works

### 2.1. Applications and Challenges of Micro-Electro-Mechanical Systems (MEMSs) in Industrial Monitoring

Micro-Electro-Mechanical System (MEMS) technology originated from semiconductor manufacturing processes, integrating mechanical structures, sensors, actuators, and electronic circuits onto a single silicon substrate through micron-scale fabrication [24]. In the field of industrial monitoring, MEMS sensors have been widely adopted for collecting multimodal signals such as vibration, inertial, acoustic, and environmental data due to their advantages of small size, low power consumption, and high reliability [25,26]. In elevator monitoring, MEMS accelerometers are used to detect guide rail irregularities and traction machine imbalance [27]; MEMS microphones can capture bearing abnormal noise and brake friction sounds [28]; Inertial Measurement Units (IMUs) can monitor car leveling accuracy and instantaneous impacts [11].

However, the deployment of MEMS perception systems in industrial environments faces multiple challenges, as MEMS devices are sensitive to environmental temperature, humidity, and mechanical stress, potentially leading to bias drift and sensitivity variations during long-term operation [29]. Secondly, weak fault signals are easily submerged by circuit thermal noise and environmental electromagnetic noise [15]. Finally, the high-frequency output of MEMS sensors requires corresponding interface and computational resources, making it difficult for traditional data acquisition systems to meet their real-time requirements [12]. The edge–cloud collaborative framework and the Elite algorithm proposed in this paper effectively address these industrial shortcomings of MEMSs.

### 2.2. Advantages and Challenges of Transformers in Industrial Time-Series Analysis

Time-series analysis models aim to handle features in data that change over time. Early approaches used traditional machine learning methods. For example, Chai et al. [30] applied Least Squares Support Vector Machines (LS-SVMs) to elevator fault identification, but they struggled with massive data volumes. Niu et al. [31] used a multi-attribute decision-making method, which failed to capture complex temporal correlations due to reliance on a single parameter type. With the development of deep learning, Recurrent Neural Networks (RNNs) [32] and their variant LSTM [19] were introduced for time-series analysis but suffer from gradient vanishing and difficulty in parallelization. Temporal Convolutional Networks (TCNs) [20] expanded the receptive field through dilated convolutions, improving long-sequence modeling capability, yet they remain limited by network depth and have limited adaptability to event-driven data. ROCKET [33], a method based on convolutional kernels, improved efficiency, but its feature extraction process is non-learnable, potentially losing subtle patterns in MEMS data.

The Transformer architecture [22], with its self-attention mechanism, can establish direct dependencies between any two points in a sequence, providing a breakthrough solution for long-sequence modeling. In the field of time-series forecasting, variants like Informer [34] and Autoformer [35] have demonstrated the advantages of Transformers in handling long-term dependencies by improving the attention mechanism to reduce computational complexity. In the elevator domain, Zhang et al. [36] applied Transformers to group supervisory prediction, optimizing elevator dispatching efficiency; Fei et al. [37] proposed MA-Transformer, which showed excellent performance in escalator passenger flow prediction.

However, applying the standard Transformer directly to industrial MEMS data faces two major challenges: The first is the high computational complexity. The quadratic computational complexity of self-attention makes it difficult to run in real-time on resource-constrained edge devices. The second is poor domain adaptability. Standard positional encoding is based on the assumption of uniform time flow, which does not align with the event-driven characteristics of industrial data [16]. Existing lightweight Transformer research, such as Lite Transformer [38] and MobileViT [39], is mainly focused on NLP or CV domains, lacking specialized designs for the inherent structure of industrial time-series data, especially MEMS data. Designing a lightweight model that retains the global modeling capability of the Transformer, adapts to the characteristics of MEMS data, and is suitable for edge deployment remains an urgent problem to be solved.

### 2.3. Application of Large Language Models and Knowledge Augmentation in Fault Diagnosis

Large Language Models (LLMs) can understand and generate natural language and, in theory, can act as fault analysis experts [40]. However, their application in specialized industrial scenarios still faces limitations. General-purpose LLMs lack fine-grained, real-time updated domain knowledge, leading to potentially inaccurate or non-actionable diagnostic suggestions [41]. Secondly, LLMs are essentially pure text models and cannot directly perceive and understand real-time, multimodal MEMS sensor data streams. The retrieval-augmented generation (RAG) framework significantly improves the accuracy and domain relevance of generated content by combining LLMs with external knowledge bases [42]. Recently, preliminary explorations have attempted to use RAG for equipment fault Q&A or maintenance instruction generation [43]. However, most of this work remains at the level of text query to text response and has not yet established a collaborative system capable of deeply fusing real-time sensor evidence chains, dynamic multimodal features, and structured domain knowledge for closed-loop diagnosis and decision-making. Enabling them not only to retrieve textual knowledge but also to understand and reason about real-time sensor data is a key challenge in achieving true intelligence in industrial operations and maintenance.

## 3. Materials and Methods

This section details the overall architecture and core implementation workflow of the proposed edge–cloud collaborative framework intelligent elevator operation and maintenance system based on MEMS-based perception. The system aims to achieve precise diagnosis and early warning of complex elevator faults through multi-MEMS sensor data fusion, lightweight intelligent detection at the edge, and knowledge-enhanced decision-making in the cloud.

### 3.1. System Architecture and Hardware Implementation

**Figure 1:**
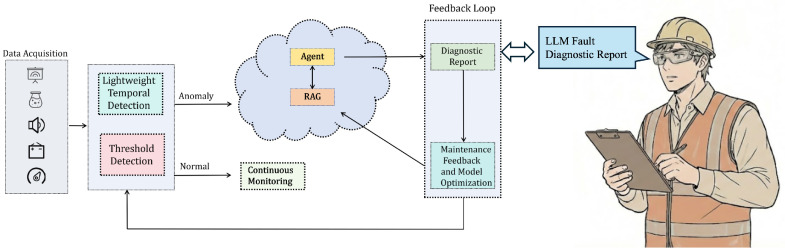
System Architecture Overview.

This study proposes an edge–cloud collaborative framework intelligent elevator operation and maintenance system with MEMS perception at its core, aiming to systematically address the three major limitations faced by MEMS sensors in industrial deployment: data throughput, signal noise, and the semantic gap. As shown in Figure 1, the proposed system transitions from static monitoring to a wearable paradigm centered on smart glasses. The device serves as a mobile perception node, where the data acquisition layer integrates multimodal sensors: a camera for visual inspection, a MEMS microphone for acoustic analysis, and an IMU for motion tracking. The edge processor performs lightweight temporal and threshold detection to identify anomalies. Crucially, the Feedback Loop can utilize the augmented reality (AR) display of the smart glasses to present the LLM Fault Diagnostic Report directly in the worker’s field of view, enabling hands-free maintenance guided by real-time expertise. These sensors feature high integration, low power consumption, and high response frequency and are suitable for embedding into the limited space of an elevator. The raw data first converge into the edge processing layer, which is centered on an NVIDIA Jetson Xavier NX (NVIDIA Corporation, Santa Clara, CA, USA). This layer handles tasks such as data synchronization, multi-scale window segmentation, and lightweight ELiTe-Transformer model inference. Its key function is the real-time preprocessing and feature extraction of high-throughput, non-uniform MEMS time-series data, and running the INT8-quantized lightweight model for millisecond-level anomaly detection and preliminary fault classification. When the edge side detects a transient fault through threshold-based detection or identifies an excessively high fault confidence output by the ELiTe-Transformer model, the system automatically triggers the cloud-side analysis process. At this point, the edge layer packages the multimodal MEMS feature evidence chain and preliminary diagnosis results into a structured evidence chain and uploads it to the cloud server. The cloud analysis layer integrates the R1-Distill-Qwen-32B and a professional knowledge base built on RAG to perform fusion reasoning and root cause analysis on the received multi-source MEMS information, generating a comprehensive diagnostic report containing specific repair suggestions. Finally, the diagnostic results are fed back to maintenance personnel in real-time through the output layer. Through this dynamic collaboration mechanism, the system fully leverages the real-time potential of MEMS perception via the edge computing architecture while compensating for its semantic output shortcomings by relying on the cloud’s knowledge enhancement capability, forming a complete technical closed loop from physical signal acquisition, edge intelligent preprocessing, and cloud knowledge-enhanced reasoning to actionable decision output.

### 3.2. Data Collection and Preprocessing

**Figure 2:**
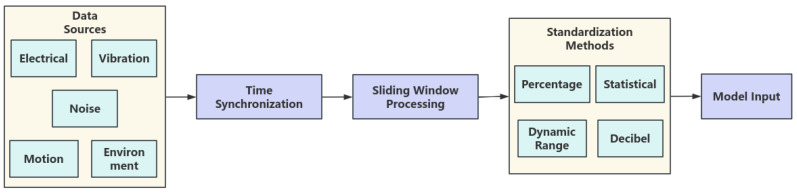
Data Acquisition and Processing Flow.

Data serves as the foundation of an intelligent diagnostic system. To achieve comprehensive perception of multi-component and multi-mode faults in elevators, this study designed and implemented a multi-source heterogeneous data collection and preprocessing scheme based on a MEMS-based perception system, covering key elevator subsystems. The overall processing workflow is illustrated in Figure 2.

#### 3.2.1. Data Collection

To establish a multimodal perception foundation for the elevator intelligent operation and maintenance system, this study designed a data collection scheme centered on MEMS sensors, focusing on five key types of data: electrical, vibration, noise, motion posture, and environmental data. All data collection was conducted on-site in collaboration with the Guangdong Special Equipment Inspection and Research Institute.

Electrical parameters were acquired using a non-invasive approach: current was measured via a CHK-100Y4 (Beijing SenShe Electronics Co., Ltd., Beijing, China) sensor, and voltage was obtained through a ZMPT107 (Nanjing Zeming Electronics Co., Ltd., Nanjing, Jiangsu, China) miniature voltage transformer connected in parallel. Vibration was sensed by an ADXL357 (Analog Devices, Inc., Norwood, MA, USA) industrial-grade MEMS three-axis accelerometer, installed at two critical locations—the bearing housing of the traction machine and the guide rail on top of the car. Its range was set to ±10 g, and data were sampled at 20 Hz. Key monitoring indicators include the root mean square value of vibration velocity (normal <1.8 mm/s, alert ≥2.3 mm/s, and fault ≥3.5 mm/s) and the peak value of acceleration envelope (early bearing fault threshold >0.12 gE). For acoustic monitoring, MEMS INMP621 microphones (TDK InvenSense, San Jose, CA, USA) were mounted on the inner wall of the car and in the middle of the hoistway. Their frequency response covered 60 Hz to 15 kHz, and the sampling rate was 1.0 MHz. The main metric is the increase in sound pressure level above the background noise: a rise exceeding 5 dB triggers an alert, while a rise above 15 dB is deemed a serious fault. Motion and attitude were captured by a ICM-20948 (TDK InvenSense, San Jose, CA, USA) 9-axis MEMS inertial measurement unit fixed at the center of the car floor. The accelerometer range was ±16 g, the gyroscope range was ±2000°/s, and the sampling frequency was 20 Hz. The horizontal inclination angle was considered normal below 1°, alert between 1° and 2°, and fault above 2°; the peak vertical vibration was normal below 0.2 m/s^2^, alert between 0.3 and 0.5 m/s^2^, and fault above 0.6 m/s^2^. Temperature and humidity were measured using SHT35 (Sensirion AG, Stäfa, Switzerland) MEMS sensors deployed inside the car and in the hoistway pit, with a sampling rate of 0.5 Hz. The normal temperature range was 20–28 °C inside the car and –10 to 50 °C in the pit; normal relative humidity was 30–70%, alert 70–85%, and fault above 85%. When temperature or humidity exceeded the equipment’s allowable range, the system issued an environmental warning. The water level was monitored by a MS5837 (TE Connectivity, Hampton, VA, USA) MEMS pressure-type sensor placed at the lowest point of the hoistway pit, with a range of 0–10 mH_2_O. The real-time water accumulation height was measured, with an alert threshold set at 10 mm and a fault threshold at 50 mm. To construct an effective fault detection model, a one-month dataset containing complete “normal → abnormal → recovery” temporal waveforms was collected, with precise annotations for the abnormal periods. To illustrate the practical deployment of the monitoring system. Table 1 presents the contribution of each sensor type in diagnosing typical elevator faults.

**Figure 3:**
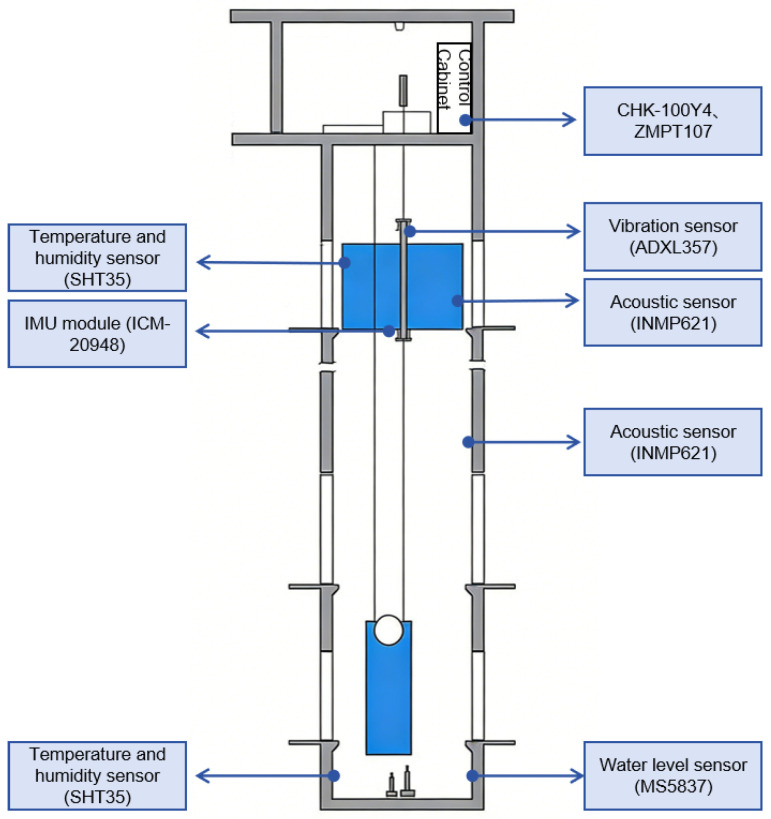
Sensor Deployment of the Proposed MEMS-Based Elevator Monitoring System.

The sensor installation locations in the elevator environment are shown in Figure 3.

#### 3.2.2. Preprocessing

To enable the fused analysis of multi-MEMS sensor data on a unified time basis, this study implemented a combined software-hardware alignment and synchronization scheme. During the collection phase, all edge collection devices were connected to the same time server via the Network Time Protocol (NTP) to calibrate their system clocks. However, due to significant differences in the sampling frequencies of different MEMS sensor data streams, direct sample point alignment was not feasible. Therefore, we constructed a unified timeline based on absolute timestamps. The raw data were parsed and converted to UTC format, virtually resampled according to the sampling period of each data stream, and assigned millisecond-level timestamps. For cross-modal analysis, logical synchronization was achieved by finding the nearest neighbor data point within a small tolerance window around the target timestamp.

**Figure 4:**
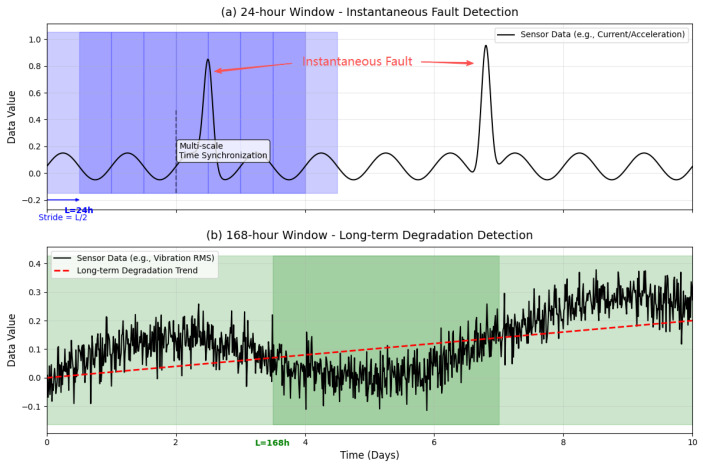
Schematic of the Multi-Scale Sliding Window Segmentation Strategy.

Synchronized continuous time-series data needed to be converted into discrete samples processable by the model. To cover fault patterns ranging from microsecond-level transient events to weeks-long gradual degradation captured by MEMS sensors, we adopted a multi-scale adaptive sliding window strategy [44], illustrated in Figure 4. This strategy dynamically configures distinct window lengths (*L*) and sliding strides (*S*) for each data stream based on the MEMS sensor type and target fault mode. Window length is key to capturing fault features: for short-term anomalies in electrical data, a 24 h window is primarily used to cover the typical daily cycle and capture sudden fault waveforms; to detect long-term performance degradation, a 168 h long window is applied in parallel to all data streams to learn subtle trends. The sliding stride controls sample overlap. Setting S=L/2 creates 50% overlap, increasing the training sample size while avoiding scenarios where brief fault events might be missed if they fall on window boundaries during inference.

The multi-scale sliding window mechanism systematically transforms the original, continuous multi-sensor data stream into a structured collection of samples with clear physical significance. This approach is particularly suited to the high-frequency, non-stationary, and multi-scale characteristics of MEMS data, laying the foundation for the lightweight time-series Transformer model to perform high-precision fault detection and diagnosis. It should be noted that sliding window segmentation and segment aggregation are edge-side data preprocessing steps completed prior to model inference. This process is mainly implemented through temporal indexing and simple statistical operations, and its computational overhead is significantly lower than that of the neural network inference phase. Therefore, the inference latency reported in the subsequent experimental section primarily reflects the single forward pass computation time of the ELiTe-Transformer model on the GPU.

#### 3.2.3. Standardization

The multi-source sensor data after window segmentation exhibit differences in units, magnitudes, and physical meanings. Directly inputting them into a model can easily lead to training bias [45]. However, industrial field data should not be standardized using generic methods; instead, it should account for the physical characteristics of MEMS sensors and the requirements of fault diagnosis. Therefore, this study proposes an adaptive standardization strategy, and corresponding methods were designed for the four categories of data: electrical, vibration, environmental, and noise.

Electrical parameters, such as voltage and current, have well-defined rated operating values, and their anomalies typically manifest as deviations from these rated values. Therefore, we employed percentage normalization based on equipment rated values. This method not only scales the data to a similar numerical range but, more importantly, preserves the key physical meaning—the degree of deviation from the nominal operating state. Its calculation formula is

(1) $$\mathrm{Standardized}\;\mathrm{value}=\frac{\mathrm{Current}\;\mathrm{measured}\;\mathrm{value}-\mathrm{Rated}\;\mathrm{value}}{\mathrm{Rated}\;\mathrm{value}}$$

Vibration acceleration data are influenced by individual equipment differences, installation positions, and environmental background vibrations. We employed dynamic normalization based on historical statistics. This method uses statistical measures from a segment of the equipment’s own historical data under normal operating conditions for standardization, aiming to eliminate individual and installation differences and highlight relative changes. Its calculation formula is

(2) $$\mathrm{Standardized}\;\mathrm{value}=\frac{\mathrm{Current}\;\mathrm{value}-\mu_{30\mathrm{day}}}{\sigma_{30\mathrm{day}}}$$

where $\mu_{30\mathrm{day}}$ and $\sigma_{30\mathrm{day}}$ are the mean and standard deviation, respectively, of the data from that sensor during normal operation over the past 30 days. Anomalies in the standardized vibration data manifest as statistical outliers.

Since attitude angles typically vary within a certain range and angular velocity is near zero during steady-state operation, we employed dynamic range normalization based on physical constraints for motion posture data:

(3) $$\mathrm{Standardized}\;\mathrm{value}=\frac{\mathrm{Current}\;\mathrm{value}-\mathrm{Physical}\;\mathrm{center}\;\mathrm{value}}{\mathrm{Maximum}\;\mathrm{allowable}\;\mathrm{deviation}}$$

Environmental parameters such as temperature, humidity, and water level usually fluctuate dynamically within a range, and their effective range may change with seasonal variations. We employed dynamic range normalization. The key aspect is that the extreme values are not fixed but are dynamically calculated and updated based on observations within a sliding time window. The formula is as follows:

(4) $$\mathrm{Standardized}\;\mathrm{value}=\frac{\mathrm{Current}\;\mathrm{value}-\mathrm{Min}\mathrm{dynamic}}{\mathrm{Max}\mathrm{dynamic}-{\mathrm{Min}}_{\mathrm{dynamic}}}$$

This method maps the data to the interval [0,1] while adapting to the long-term slow drift and seasonal changes in environmental parameters.

For the 1.0 MHz high-speed PDM bitstream output by the digital MEMS microphone, the Raspberry Pi first performed downsampling and low-pass filtering on the PDM signal to restore it to a PCM linear pulse signal x(n) so as to obtain the original time-domain waveform reflecting the sound field fluctuations inside the elevator car. The root mean square (RMS) value of the signal was calculated within a sliding analysis window of 20 ms and mapped to the logarithmic space to obtain the current relative sound pressure level $L_{p}$:

(5) $$L_{p}=20\cdot \log_{10}\left(\frac{\mathrm{RMS}(x(n))}{{\mathrm{Ref}}_{\mathrm{FS}}}\right)$$

where ${\mathrm{Ref}}_{\mathrm{FS}}$ is the full-scale reference value of the sensor. To define the effective abnormal range of the on-site environment, we designed a standardized method with dynamic range constraints. The abnormal monitoring index η was calculated by determining the on-site measured silence baseline value and safety upper limit value:

(6) $$\eta =\frac{L_{p}-\mathrm{Silence}\;{\mathrm{baseline}}_{\mathrm{dB}}}{\mathrm{Safety}\;\mathrm{upper}\;{\mathrm{limit}}_{\mathrm{dB}}-\mathrm{Silence}\;{\mathrm{baseline}}_{\mathrm{dB}}}$$

when η approaches 1, it indicates that the noise is close to the tolerance upper limit; when η> 1, a serious abnormality is immediately determined, and an early warning is triggered.

### 3.3. Fault Detection

**Figure 5:**
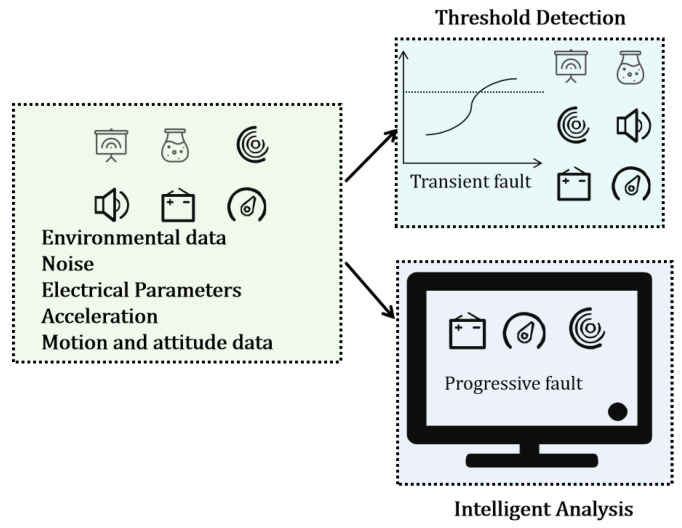
Core Architecture of the Edge-Based Intelligent Fault Detection Engine.

At the edge, the fault detection module includes a hybrid strategy to balance detection real-time performance and fault complexity. This strategy consists of two parallel branches: threshold detection is used to process sensor data that presents intuitive transient faults as well as relatively slow variations whose fault patterns can be directly defined by physical limits. The lightweight time-series detection module is specifically designed to identify long-term trend faults characterized by complex dynamic evolution, primarily processing time-series data from electrical, motion attitude, and vibration MEMS sensors, as shown in Figure 5.

#### 3.3.1. Industrial Positional Encoding

Before data is input into ELiTe-Transformer, temporal positional information needs to be added to the multi-dimensional features. However, standard positional encoding does not align with the actual characteristics of MEMS sensor data in industrial scenarios. During elevator operation, key signal data from MEMS sensors is easily submerged by background noise from normal operation. To address this, we propose an industrial positional encoding mechanism, specifically designed for the noise characteristics and event-driven nature of MEMS sensors in industrial environments, aiming to enhance sensitivity to weak features such as micro-vibrations and micro-acoustics. It additionally defines a weight function W(t), which assigns higher weights near predefined key time points to reduce contamination from background noise. The final position-aware feature $Z^{\prime }$ is calculated as follows:

(7) $$Z^{\prime }=W\left(t\right)\cdot \mathrm{StandardPE}\left(t\right)+\mathrm{Feature}\left(t\right)$$

where StandardPE(t) is the standard position encoding, Feature(t) is the feature vector at that time, and W(t) is the temporal weight. We employed a Gaussian kernel function to construct this weight distribution

(8) $$W\left(t\right)=1+\alpha \exp\left(-\frac{{\left(t-t_{\mathrm{key}}\right)}^{2}}{2\sigma^{2}}\right)$$

where $t_{\mathrm{key}}$ denotes the critical time point, σ controls the width of the weight peak, determining the influence range of the critical time point, and α controls the height of the weight peak, i.e., the maximum amplification amplitude. After calculation, the model’s weight peak for the recent fault window ≈2.2, the predictive 24 h window weight peak ≈1.8, and the long-term trend 168 h window weight peak ≈1.5.

The key time points $t_{\mathrm{key}}$ were determined based on domain knowledge and historical fault statistics, including recent anomaly periods, short-term prediction horizons, and long-term degradation windows. The parameters α and σ were empirically set and fixed across all experiments to avoid overfitting.

The industrial positional encoding mechanism endows the model with physically interpretable attention. By explicitly encoding key events in elevator operation as prior knowledge into temporal weights, the model aligns its analytical focus with actual physical processes, and subsequent diagnostic conclusions can be directly correlated with specific operational events.

#### 3.3.2. Output and Decision Mechanism

**Figure 6:**
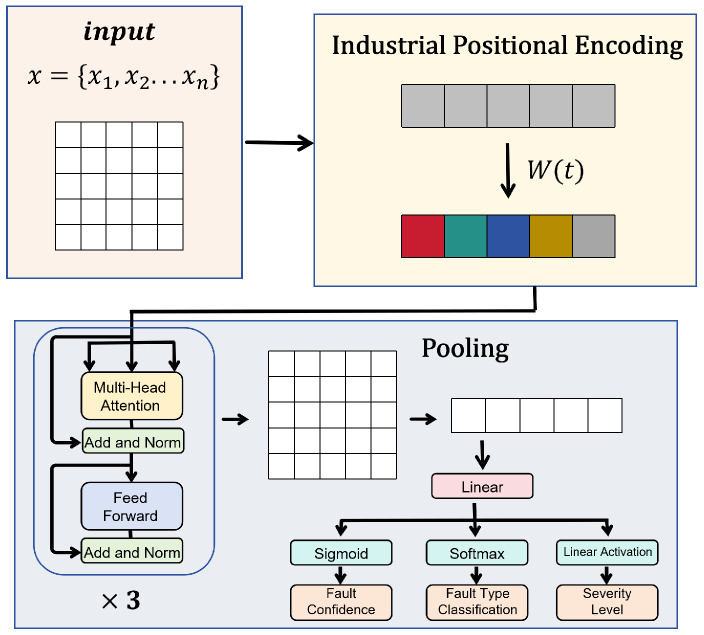
Model Architecture of the Edge-Lightweight Temporal ELiTe-Transformer.

The edge-based mechanism must provide anomaly detection and supply structured diagnostic data for subsequent cloud-based deep analysis. To this end, we designed a multi-task output head for each edge model and established a clear edge–cloud collaborative framework trigger decision mechanism. At the edge, we employed the ELiTe-Transformer temporal detection framework. This framework takes multimodal feature sequences as input, utilizing multi-layer self-attention mechanisms to model dynamic dependencies across time steps. The encoder performs the following core transformations on the input sequence:

(9) $$\begin{array}{cc} Z^{\left(l\right)} & =\mathrm{LN}\left(H^{\left(l-1\right)}+\mathrm{MHSA}\left(H^{\left(l-1\right)}\right)\right)\\ H^{\left(l\right)} & =\mathrm{LN}\left(Z^{\left(l\right)}+\mathrm{FFN}\left(Z^{\left(l\right)}\right)\right) \end{array}$$

where MHSA denotes the multi-head self-attention mechanism for capturing cross-temporal correlations; FFN represents the feedforward neural network; and LN signifies the layer normalization operation. After stacking L layers, the model acquires a global temporal context representation $H^{\left(l\right)}$. The model ultimately completes three inference tasks through pooling layers combined with multi-task output heads: (1) fault confidence prediction $p_{f}=\sigma \left(W_{f}\bar{H}\right)$, (2) fault type classification $P_{c}=\mathrm{Softmax}\left(W_{c}\bar{H}\right)$, and (3) severity-level regression $s=W_{s}\bar{H}$, where $\bar{H}$ denotes the sequence-level representation after global average pooling. Instead of outputting a single, unintelligible anomaly score, the multi-task output head directly decomposes high-dimensional MEMS features into three dimensions with explicit engineering implications: fault confidence, fault type and severity. This structured output serves as a preliminary, quantifiable interpretation of equipment health status. The overall model structure is illustrated in Figure 6.

**Figure 7:**
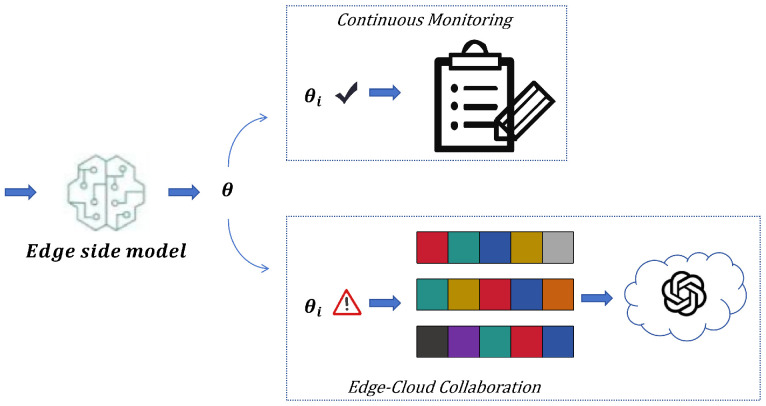
Edge–Cloud Collaborative Framework Decision-Making Flowchart.

After processing the sliding window data, the ELiTe-Transformer model obtains a sequence-level feature representation through global average pooling, which is then fed into a triple-branch output head for fault confidence, fault type, and severity level. When the fault confidence of any edge model exceeds the threshold θ, the system immediately aggregates the multimodal features and diagnostic results from all MEMS sensors within the current window to form a state snapshot, which is then uploaded to the cloud for analysis. The decision workflow is illustrated in Figure 7. The cloud-based agent performs fusion reasoning based on this multimodal chain of evidence to generate a comprehensive diagnostic report.

#### 3.3.3. Lightweight Design

To meet the stringent constraints of industrial edge devices regarding memory, computational power, and energy consumption, as well as the need to process high-throughput data streams from multi-channel MEMS sensors in real-time, we implemented comprehensive lightweight design. This approach significantly reduces model complexity and computational overhead while maximizing the retention of its representational capabilities for ELiTe-Transformer.

**Architectural Compression:** We achieve foundational lightweighting through precise pruning across model dimensions: reducing the hidden layer dimensions from the standard model’s $d_{model}=512$ to 128 and decreasing multi-head attention heads from h=8 to 4—limiting the encoder stack depth from L=4 to 3. This multi-dimensional pruning reduces the model’s baseline computational load.

**Efficient Attention Mechanism:** A linear attention variant addresses computational bottlenecks in long sequences. The quadratic computational complexity of standard attention reduces ($O(n^{2})$) to linear complexity (O(n)). The core formula transformation is as follows:

(10) $$\mathrm{LinearAttention}\left(Q,K,V\right)=\frac{\phi \left(Q\right)\left(\phi {\left(K\right)}^{T}V\right)}{\phi \left(Q\right)\left(\phi {\left(K\right)}^{T}1\right)}$$

where ϕ(·) typically represents simple non-linear functions such as elu(·)+1, resolving computational bottlenecks in long sequence processing.

**Feedforward Network Optimization:** Bottleneck design was employed to reduce the FFN parameter count, significantly lowering the expansion factor of the feedforward layer. The GeLU activation function was selected to enhance training stability.

**Model Quantization:** Post-training static quantization was applied, mapping weights and activation values from FP32 to INT8:

(11) $$x_{\mathrm{int}8}=\mathrm{round}\left(\frac{x_{\mathrm{fp}32}-\mathrm{offset}}{\mathrm{scale}}\right)$$

where $\mathrm{scale}=\frac{\max(x)-\min(x)}{2^{8}-1}$, preserving FP16 precision for sensitive layers while quantizing computationally intensive layers to INT8, reducing model size by approximately 75%.

### 3.4. Cloud-Based Deep Analysis and Knowledge Augmentation

**Figure 8:**
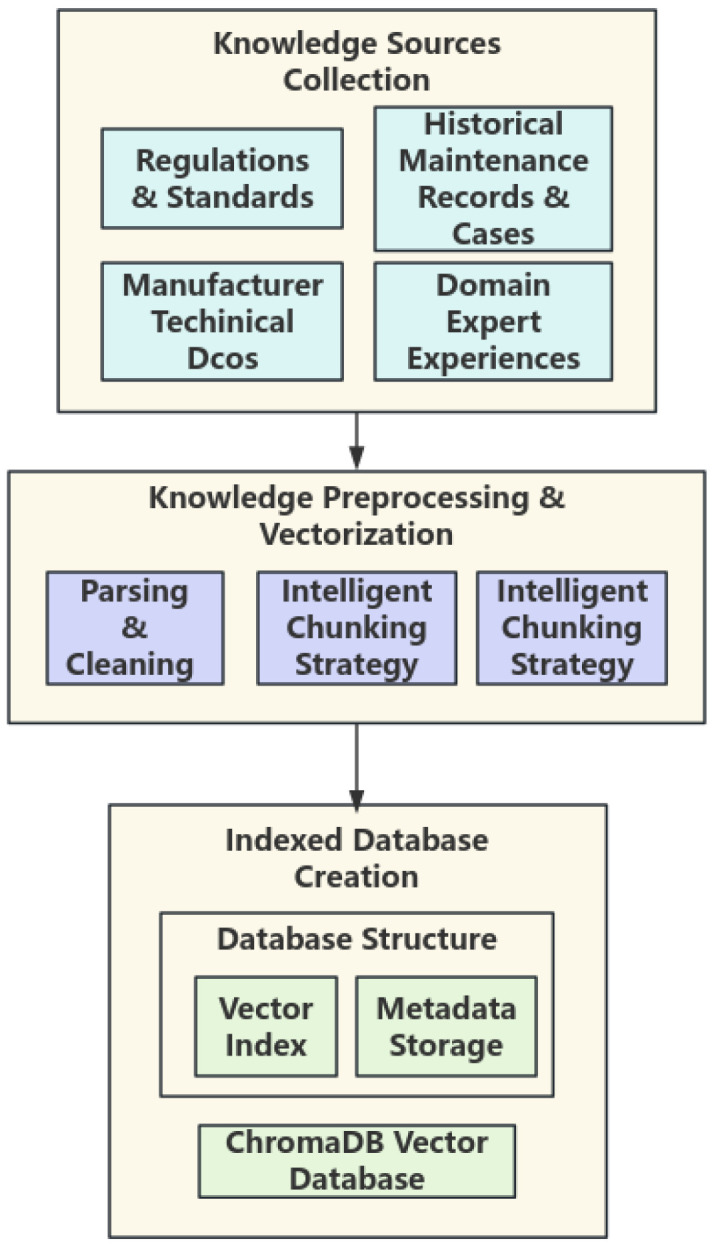
RAG Knowledge Base Construction Flowchart.

The cloud module receives MEMS sensor feature data and preliminary diagnostic results uploaded from the edge side. Leveraging a professional knowledge base constructed with RAG technology, it performs deep fusion reasoning and root cause analysis, ultimately transforming the low-semantic information from MEMS sensors into interpretable and actionable diagnostic reports. The overall process is illustrated in Figure 8.

The knowledge base integrates multi-source professional knowledge covering the entire lifecycle of elevator operation and maintenance, including regulations and standards, manufacturer technical documentation, historical repair cases, and expert experience summaries. These heterogeneous documents are converted into text chunks of 512–1024 characters in length through parsing, cleaning, and a recursive segmentation strategy based on chapter structure. Subsequently, the text-embedding-ada-002 model is used to vectorize each text chunk into a 1536-dimensional semantic vector, which is stored in a vector database to enable efficient similarity retrieval. This knowledge base provides the cloud-side agent with precise, traceable domain knowledge support.

**Figure 9:**
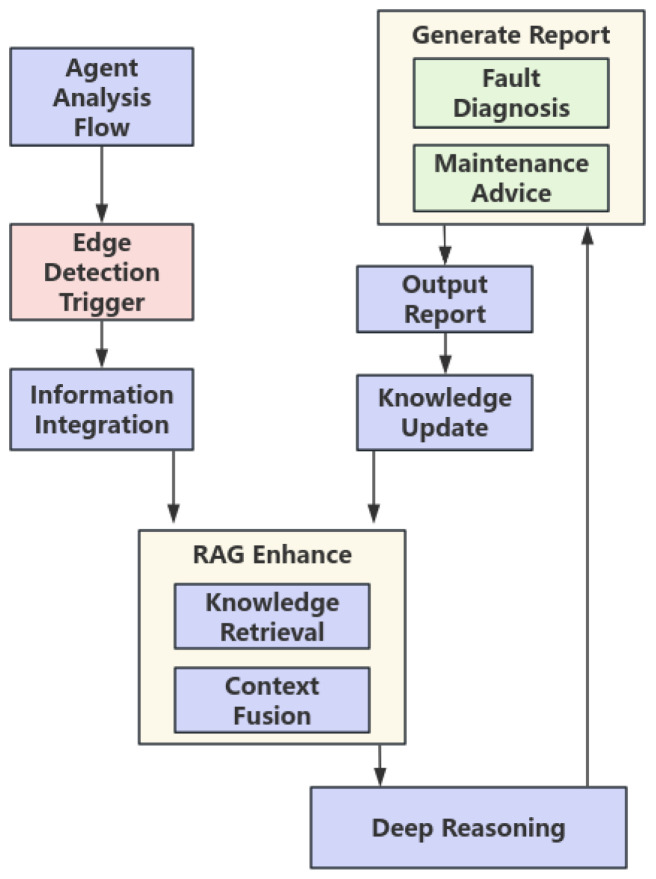
Cloud-Based Large Model Agent Analysis Flowchart.

As illustrated in Figure 9, when an anomaly is triggered at the edge side, the agent receives and integrates the multimodal context, automatically generates a targeted query, and retrieves relevant professional knowledge snippets from the RAG. Subsequently, R1-Distill-Qwen-32B fuses the real-time evidence with the retrieved knowledge to perform multi-step reasoning, completing fault confirmation, root cause analysis, risk assessment, and repair suggestion generation. Finally, it outputs a structured comprehensive diagnostic report that helps understand the physical meaning behind the MEMS data.

Furthermore, this system possesses a closed-loop learning capability, enabling continuous optimization of the model and knowledge base based on actual operation and maintenance feedback. Maintenance work orders are automatically analyzed weekly to identify misjudged cases. The corresponding MEMS data and diagnostic logs from those periods are extracted to build a correction sample library for incremental model training. Successfully repaired cases, after review, are structured and stored in the RAG knowledge base. The system also supports dynamic updates and revisions to the knowledge base content, ensuring its accuracy and timeliness evolve over time. This mechanism allows the system to learn continuously from practice, steadily improving diagnostic reliability and decision quality.

This system includes RAG technology to annotate statistical features of MEMS data with physical and engineering connotations. The cloud agent does not conduct black-box inference relying on its own parameters; instead, it takes the feature evidence chain extracted at the edge as a query to perform precise retrieval in a structured knowledge base covering industry standards, technical manuals and maintenance cases. Every conclusion in the diagnostic report is traceable to specific feature data and domain knowledge, which guarantees the report’s traceability and verifiability, as well as the interpretability of the system.

## 4. Results

### 4.1. Experimental Environment and Dataset

To validate the effectiveness of the proposed edge–cloud collaborative framework intelligent operation and maintenance system for elevators based on MEMS-based perception, we established a real experimental environment and constructed an evaluation dataset based on multi-MEMS sensor data collected from elevator sites. The edge side of the system was deployed on an NVIDIA Jetson Xavier NX (NVIDIA Corporation, Santa Clara, CA, USA) platform, configured with a 6-core ARM v8.2 64-bit CPU, a 384-core Volta GPU, and 8GB LPDDR4x memory. The inference framework used was TensorRT 8.4, with model precision set to INT8 quantization. The cloud-side agent was deployed on a server equipped with an NVIDIA A100 GPU used to run the R1-Distill-Qwen-32B large language model and the vector retrieval service for the RAG knowledge base. The software environment utilized Python 3.8, PyTorch 1.12, Transformers 4.20, ChromaDB, etc.

**Figure 10:**
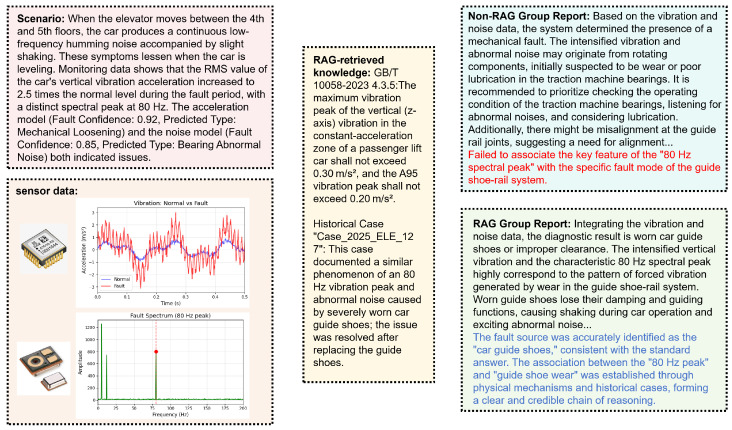
Schematic Diagram of an Elevator Fault Diagnosis Example Scenario Analysis.

For model training and performance evaluation, we collected continuous operational data over 6 months via deployed MEMS-based accelerometers, microphones, IMUs, and environmental sensors. Approximately 50,000 valid multi-scale sliding window samples were extracted. These were split using a time-based approach into training, validation, and test sets in a 7:2:1 ratio. The final test set contained 2000 samples covering five major categories and a total of 12 specific faults, including bearing wear, abnormal motor current, door mechanism jamming, guide shoe aging, and hoistway water ingress. We selected models widely used in industrial time-series analysis, LSTM [19], 1D-CNN [19], TCN [20], and Transformer [22], as baselines. All models were trained on the same training set using the AdamW optimizer and cross-entropy loss function. The optimal number of training epochs was determined via an early stopping strategy on the validation set to ensure a fair comparison of edge-side model performance. To specifically test the collaborative diagnostic capability of the cloud-side agent, we further delineated a complex fault subset containing 200 scenarios from the test set. The construction of the complex fault subset is based on two criteria: (i) faults involving multiple subsystems that require cross-sensor correlation for accurate diagnosis and (ii) faults with weak features in a single sensor channel, identified by domain experts based on historical maintenance logs. In practice, we first extracted all samples labeled as known multi-source or weak fault types from the test set, such as early-stage bearing faults and intermittent door jamming. Subsequently, three senior elevator maintenance engineers reviewed these samples to confirm that their correct diagnosis integrated information from at least two different MEMS sensor modalities. We designed three sets of comparative experiments: Group A (No Collaboration) relied solely on the independent detection results of each edge lightweight model, simulating traditional decentralized MEMS monitoring solutions. In Group B (Pure LLM), raw multi-sensor data were fed directly into the R1-Distill-Qwen-32B for feature extraction and fault diagnosis without leveraging edge model preprocessing. Specifically, we converted each sliding window sample into a structured textual prompt. For a given time window, the data from each MEMS sensor was listed in chronological order, presented in a comma-separated format, along with the corresponding timestamps. Group C (proposed system) employed the edge–cloud collaborative framework architecture proposed in this paper, where the small edge model output preliminary detection results and multimodal features, and the large cloud model agent performed comprehensive diagnosis and knowledge-enhanced reasoning. Finally, we conducted a comparison experiment with and without the professional elevator knowledge RAG enhancement for R1-Distill-Qwen-32B to verify the contribution of the RAG knowledge base in improving the accuracy, professionalism, and reliability of the cloud-side agent’s diagnostic reports. We first constructed a test set containing 200 fault scenarios, of which 100 scenarios’ diagnostic conclusions heavily relied on external professional knowledge, and the remaining 100 were relatively general problems. An example of fault analysis is illustrated in Figure 10 by our system.

### 4.2. Implementation of the Context-Based Evaluation Framework

In practical applications of industrial inspection equipment, the complex field environments and variable sensor operating conditions often render single global accuracy metrics inadequate for objectively assessing a system’s true efficacy. Particularly when expanding the research perspective to personal inspection or wearable sensing domains, a system’s adaptability to complex semantic contexts becomes critical. To address this, a structured context-based evaluation framework was employed in this study, as detailed in the experimental validation section. Context is explicitly defined as a comprehensive semantic background comprising process stages, spatial environment, signal quality, and network status.

At the operational level, this framework ensures rigorous evaluation through three core dimensions. First, a context-aware preprocessing and calibration mechanism dynamically switches normalization strategies based on perceived environmental contexts. This eliminates distribution shifts caused by sensor adhesion variations or environmental fluctuations, ensuring physical comparability of signals across different inspection sites. Second, the system exhibits high contextual sensitivity. Edge models dynamically adjust the frequency of edge–cloud collaborative framework based on classification confidence and current network link status, prioritizing decision continuity under extreme operating conditions. Finally, for validation, we employed a strict time-based split approach to simulate context drift during actual inspection cycles, thereby testing the model’s generalization robustness against sensor aging or operational state transitions.

Based on this framework, we established a multidimensional metric system to evaluate the system’s application value from various perspectives. We used edge-side inference latency and memory consumption metrics to measure the algorithm’s real-time responsiveness on portable inspection devices. Comprehensive classification performance on the time-series split test set was used to evaluate the system’s reliability when handling operational condition shifts. Additionally, evaluation tools tailored for large language models were employed to assess the practical guidance value of the system’s generated diagnostic reports for inspection personnel, focusing on factual consistency and context recall rates. Through this multidimensional mapping, system evaluation transitions from purely verifying algorithmic accuracy to comprehensively considering decision-making usability within complex industrial scenarios.

### 4.3. Efficient Processing of MEMS Data by the ELiTe-Transformer Edge Model

To verify the capability of the ELiTe-Transformer model to efficiently process high-frequency, high-dimensional MEMS data streams on resource-constrained edge devices, this section presents a comprehensive comparison of its performance and efficiency against various classical time-series models.

The experimental results, as detailed in Table 2, show that in terms of diagnostic accuracy, the proposed ELiTe-Transformer model achieves performance comparable to the strongest baseline, the TCN, and is significantly better than LSTM and the 1D-CNN. This demonstrates its powerful ability to model complex temporal patterns in MEMS data while being lightweight. Regarding efficiency metrics critical for edge deployment feasibility, through architectural compression and quantization, the model size is compressed to 9.8 MB, approximately 20.1% of the standard Transformer model’s size. An inference latency of 21.4 ms indicates that the model can process data streams at a frequency exceeding 50 Hz, fully meeting the real-time monitoring requirements for high-frequency MEMS sensors. For further clarification, inference latency refers to the average time taken by the model to perform one forward inference on a single sliding window sample on the Jetson Xavier NX GPU. This metric only reflects the latency of the model computation stage, excluding system-level overheads such as sensor data acquisition, sliding window segmentation, and edge-cloud communication. Although the 1D-CNN achieves lower latency due to the parallel nature of local convolutions, its accuracy lags behind. ELiTe-Transformer achieves a balance between accuracy and efficiency for high-frequency MEMS data streams, specifically addressing the conflict between high data throughput of MEMSs and the limited computing power of edge devices.

### 4.4. Ablation Study

To validate the key designs in ELiTe-Transformer that address the characteristics of MEMS sensor industrial applications, we conducted an ablation study. The results are shown in Table 3.

Compared to the standard positional encoding, the industrial positional encoding improved the F1 score by 1.2%, demonstrating that this mechanism can effectively address the challenge of key signals in MEMS data being submerged by background noise. By emphasizing weights around key time points, industrial positional encoding enhanced the model’s sensitivity for fault detection in noisy industrial environments. The linear attention mechanism significantly reduced inference latency from 185.6 ms to 21.4 ms, achieving an 8.7× speedup, which is key to enabling the model to process long MEMS sequences in real-time at the edge. The performance of models using a single window length declined, while the multi-scale window strategy achieved an F1 score 1.5% higher than the best single-scale window, verifying its ability to simultaneously capture both transient anomalies and long-term degradation trends in MEMS data. INT8 quantization reduced the model size by 75% and decreased inference latency by 68.8%, with only a 0.1% loss in accuracy, greatly enhancing the model’s deployment feasibility on resource-constrained edge hardware.

### 4.5. The Cloud-Side Large Model Agent Bridges the Semantic Gap

To validate the value of the edge–cloud collaborative framework architecture in bridging the semantic gap between MEMS data and operation and maintenance decisions, we conducted tests on a complex fault subset. Based on 300 event samples of faults partially identifiable by single sensors—such as bearing wear, motor current surge, and door mechanism jamming—we designed complex, multi-source fault scenarios encompassing cross-sensor correlations, subtle precursors, and multi-system coupling, totaling 200 scenarios. All samples were manually annotated by domain experts, including primary fault type, component-level root cause, and fault onset time.

As shown in Table 4, in terms of diagnostic accuracy and root cause identification rate, Group C significantly outperforms both Group A and Group B. This demonstrates that the systematic solution—comprising real-time preprocessing and feature extraction of raw MEMS data by the edge ELiTe-Transformer, followed by deep reasoning performed by the cloud-side agent through fusion of multimodal evidence chains and domain knowledge—can effectively overcome the limitations of single-sensor or single-model capabilities. In complex fault scenarios characterized by weak features and requiring cross-sensor correlation, the edge–cloud collaborative framework demonstrates substantial value.

As shown in Table 5. For rare faults, the proposed system (Group C) achieves significantly higher F1 scores compared to the other two groups. On complex faults with more subtle features and relatively scarce samples, our method is able to correlate multi-source MEMS information and conduct deep root cause analysis, successfully transforming low-semantic sensor readings into high-value diagnostic insights.

### 4.6. Contribution Analysis of RAG Knowledge Enhancement

To further verify how the RAG mechanism transforms MEMS physical features into understandable maintenance decisions, we evaluated its impact on the output quality of the cloud-side agent. The results are shown in Table 6:

In this study, if a specific statement in the generated report lacked clear supporting evidence in the corresponding RAG retrieval results, equipment technical manuals, or historical fault cases, or if it contradicted the conclusions annotated by field experts, the statement was determined to be hallucinated. The hallucination rate was calculated using a semi-automated evaluation process, with the specific steps as follows: First, the diagnostic report generated by the model was split into several atomic statements. Each statement was then compared against the knowledge segments retrieved by RAG. If the key information in a statement could be supported by any retrieved segment or if it contradicted the expert-annotated conclusions, it was marked as a hallucination. The hallucination rate was defined as the proportion of sentences marked as hallucinations among the total number of sentences in the report.

From the perspective of the resulting data, we used the Ragas framework [46] to automatically evaluate the professionalism of diagnostic reports. The RAG mechanism provided the agent with precise and reliable domain knowledge anchors, significantly improving the diagnostic professionalism score by 30.9%. Examining the component metrics, Faithfulness increased by 43.1%, and the hallucination rate was reduced by 71.4%. This indicates that by correlating the microscopic physical features detected at the edge with industry standards, technical manuals, and historical cases in the knowledge base, RAG effectively endows MEMS data with accurate physical meaning and maintenance context, fundamentally bridging the semantic gap between the MEMS-based perception system and final business decisions.

## 5. Discussion

### 5.1. In-Depth Analysis of Ablation Experiments

Ablation experiments reveal the contribution of each key module to system performance. To further explore the working mechanism of each module, we conducted a hierarchical analysis of model performance across different fault types. Industrial positional encoding improves the F1 score by 1.2% over standard positional encoding. A fault-type breakdown shows this mechanism delivers the most significant gains for weak-feature faults (2.3% for early bearing wear and 1.9% for minor guide shoe wear) while improving strong-feature faults such as current anomalies by only 0.4%. This confirms that assigning higher weights to critical time points via Gaussian kernel functions enhances the model’s sensitivity to weak event-driven features submerged in background noise, demonstrating robustness to low signal-to-noise ratio conditions.

Linear attention reduces inference latency to 1/8.7 with only a 0.2% drop in the overall F1 score. Further analysis indicates that performance loss mainly occurs in transient faults lasting less than 1 s, likely because linear attention is inferior to standard attention in modeling local fine-grained temporal relationships. Nevertheless, it maintains accuracy comparable to standard attention for most elevator faults, verifying its suitability for edge deployment scenarios.

The multi-scale window strategy increases the F1 score by 1.5% over the single 24 h window. Analysis shows the 24 h window achieves higher detection accuracy for sudden faults, while the 168 h window provides stronger early warning for progressive degradation faults. Their fusion enables the system to capture both transient anomalies and long-term trends, achieving full coverage of fault types.

INT8 quantization shrinks the model size by 75% with only a 0.1% accuracy loss. Error analysis reveals quantization errors concentrate on low-SNR samples, pointing to future optimization directions: retaining FP16 precision for noise-sensitive layers or adopting quantization-aware training.

### 5.2. Interpretability Analysis

To evaluate the system’s generalization under varying conditions, we further analyzed model performance across different fault types, noise environments and elevator models. For common faults such as bearing wear and current anomalies, the system F1 score exceeds 92%; for complex coupled faults with limited samples, it still maintains an 89.8% root cause identification rate, proving the RAG mechanism compensates for training data sparsity by retrieving similar historical cases. For noise robustness, industrial positional encoding was designed to address the signal-to-noise ratio challenges of MEMS sensors in industrial environments, thus enhancing fault detection with strong background noise. The 1.2% F1 gain in Table 3 validates the effectiveness of this mechanism on real industrial data. In addition, the proposed method is model-agnostic: the lightweight ELiTe-Transformer architecture and the RAG knowledge base can be transferred to other elevator models or rotating machinery (e.g., fans and pumps), requiring only fine-tuning of standardization parameters and expansion of the knowledge base. Preliminary cross-domain tests show that the model achieves 82.3% zero-shot diagnosis accuracy on another brand of elevators without fine-tuning, which rises to 94.1% after fine-tuning with a small number of samples.

### 5.3. System Generalization Capability Analysis

The industrial positional encoding mechanism aligns the model’s attention on time series with key physical events in elevator operation through a Gaussian weight function, giving the model’s enhanced sensitivity to fault signals clear physical meaning. The multi-task output head directly maps complex sensor data to three engineering-semantic dimensions: fault confidence, fault type and severity, completing the structured translation from data to preliminary diagnostic information. The retrieval-augmented generation mechanism takes low-level features extracted at the edge as retrieval cues to match each feature with corresponding domain knowledge annotations from the knowledge base consisting of industry standards and technical manuals.

The final comprehensive diagnostic report integrates all the above interpretive information, which explicitly includes evidence (edge feature chain), basis (knowledge retrieved via RAG) and recommendations (maintenance solutions).

### 5.4. Limitation Analysis

Despite the satisfactory performance, the system still has several limitations. First, the validation of model generalization is limited. Elevators from different manufacturers, production years and load capacities feature large differences in vibration transmission characteristics and fault feature distributions, requiring further large-scale cross-site and cross-model validation. Second, the current system includes an offline training mode and cannot adapt online to sensor aging, environment drift or the emergence of new fault modes. Although the RAG knowledge base supports dynamic updates, the edge model itself lacks continual learning capability, and online or incremental learning mechanisms will be introduced in the future. Third, the retrieval performance of the RAG module highly depends on the quality and coverage of the knowledge base. The knowledge base constructed in this study is mainly based on public standards, technical manuals and historical work orders, and its support for rare or new faults needs verification. Fourth, the stability of the edge–cloud collaborative framework mechanism in weak-network environments has not been fully tested. When the network is interrupted, cloud analysis functions degrade, and the system can only rely on preliminary edge diagnosis results, which may affect the accurate identification of complex faults.

## 6. Conclusions

To address the three core challenges faced by MEMS sensors in elevator fault diagnosis—data processing, noise interference, and the semantic gap—this study proposes an intelligent edge–cloud collaborative framework diagnostic framework. The key insight of this study is that unlocking the industrial value of MEMS data relies not only on advanced algorithms but also on the systemic reconstruction of the “sensing-computing-decision” chain. Based on this, we designed the ELiTe-Transformer model for the edge side—a novel lightweight temporal model that enhances the capture of weak features from a physical perspective through industrial positional encoding, while adapting to the hardware constraints of edge devices at the architectural level via linear attention and quantization techniques. Experiments show that the model achieves 96.0% accuracy with a low latency of 21.4 ms on the Jetson platform. Furthermore, we innovatively introduced a RAG mechanism to align underlying physical features with high-level operational knowledge for the first time: by transforming MEMS physical features into structured maintenance knowledge, the semantic gap between low-level numerical data and high-level decision-making is bridged, enabling the transformation from data observation to fault understanding. Within the edge–cloud collaborative framework, the accuracy of complex fault diagnosis is improved to 92.5%, and RAG significantly enhances the professionalism of the generated reports. The experimental results validate the system’s superiority in accuracy, real-time performance, and interpretability. This study provides a feasible paradigm for the deep application of MEMS perception in industrial operations and maintenance. Future work will focus on: (1) improving model generalization across diverse elevator configurations for MEMS plug-and-play; (2) online learning to accommodate device aging and emerging fault patterns; (3) extending MEMS multimodal inputs for enhanced early warning; and (4) federated learning for privacy-preserving collaborative diagnosis across large-scale deployments.

## Figures and Tables

**Table 1:** Sensor-Fault Diagnosis Capability Matrix (• core sensor, ∘ auxiliary sensor, - not involved).

Sensor Type	Bearing Wear	Guide Rail Irregularity	Door Jamming	Current Imbalance	Pit Water Accumulation	Rope Slippage
Vibration sensor (ADXL357)	•	∘	-	∘	-	-
Motion attitude sensor (ICM-20948)	-	•	-	-	-	•
Noise sensor (INMP621)	∘	-	•	-	-	∘
Electrical parameter sensor (CHK-100Y4 + ZMPT107)	∘	-	∘	•	-	∘
Temperature and humidity sensor (SHT35)	-	-	-	∘	∘	-
Water level sensor (MS5837)	-	-	-	-	•	-

**Table 2:** Performance and Efficiency Comparison Between ELiTe-Transformer and Baseline Models.

Model	Accuracy (%)	Recall (%)	F1 Score (%)	Model Size (MB)	Inference Latency (ms)	Computational Load (GFLOPs)
LSTM	93.5	91.8	92.6	3.2	45.2	0.38
1D-CNN	95.1	94.3	94.7	4.1	12.5	0.05
TCN	96.2	95.7	95.9	8.7	28.9	0.21
Transformer	96.3	95.8	96.0	48.7	180.0	8.9
Ours	96.0	95.2	95.6	9.8	21.4	0.32

**Table 3:** Ablation Study Results of ELiTe-Transformer.

Configuration	Accuracy (%)	Recall (%)	F1 Score (%)	Model Size (MB)	Inference Latency (ms)
Full model (ELiTe-Transformer)	96.0	95.2	95.6	9.8	21.4
Standard positional encoding	94.8	94.1	94.4	9.8	21.2
Standard attention	95.8	95.0	95.4	9.8	185.6
Single-scale window (24 h)	94.5	93.7	94.1	9.8	10.8
Single-scale window (168 h)	93.2	92.5	92.8	9.8	75.3
FP32 precision (no quantization)	96.1	95.3	95.7	39.2	68.5
FP16 precision	96.0	95.2	95.6	19.6	35.2

**Table 4:** Comparative Performance of Cloud-Based Large Model Agent Collaborative Diagnosis.

Experimental Group	Diagnostic Accuracy (%)	Root Cause Identification Rate (%)	End-to-End Latency (ms)
Group A	78.3	70.2	18.7
Group B	85.1	80.5	2850.4
Group C	92.5	89.8	152.3

Group A: No Collaboration; Group B: Pure LLM; Group C: proposed system.

**Table 5:** Comparison of Precision, Recall, and F1 Score (%) for Different Fault Categories (partial).

Fault Categories	Experimental Group	Precision (%)	Recall (%)	F1 Score (%)
Bearing Wear	Group A	81.5	75.2	78.2
	Group B	88.3	83.6	85.9
	Group C	93.1	91.5	92.3
Abnormal Motor Current	Group A	85.4	88.9	87.1
	Group B	90.2	87.5	88.8
	Group C	94.7	92.1	93.4

Group A: No Collaboration; Group B: Pure LLM; Group C: proposed system.

**Table 6:** Automated Evaluation Results for RAG Knowledge Enhancement Effects.

Evaluation Metric	Detailed Regulations	Group A (Without RAG)	Group A + RAG (Proposed System)
Ragas [46]	Ragas Score	0.68	0.89
	Faithfulness	0.65	0.93
	Context Recall	0.68	0.88
	Answer Relevance	0.72	0.85
1—FactCC score [47]		0.21	0.06
Response relevance (1–5)		3.9	4.7
Completeness (1–5)		3.5	4.6

## Data Availability

The data presented in this study are available on request from the corresponding author due to commercial confidentiality.

## Abbreviations

The following abbreviations are used in this manuscript:

MEMS	Micro-Electro-Mechanical Systems
IIoT	Industrial Internet of Things
RAG	Retrieval-Augmented Generation
LLM	Large Language Model
ELiTe-Transformer	Efficient Lightweight Time-Series Transformer
IMU	Inertial Measurement Unit
CNN	Convolutional Neural Network
LSTM	Long Short-Term Memory
TCN	Temporal Convolutional Network
SVM	Support Vector Machine
FFN	Feed-Forward Network
MHSA	Multi-Head Self-Attention
LN	Layer Normalization
NTP	Network Time Protocol
UTC	Coordinated Universal Time
GPU	Graphics Processing Unit
CPU	Central Processing Unit
DLA	Deep Learning Accelerator
INT8	8-Bit Integer
FP32	32-Bit Floating Point
FP16	16-Bit Floating Point
